# E2F7 Transcriptionally Inhibits MicroRNA-199b Expression to Promote USP47, Thereby Enhancing Colon Cancer Tumor Stem Cell Activity and Promoting the Occurrence of Colon Cancer

**DOI:** 10.3389/fonc.2020.565449

**Published:** 2021-01-07

**Authors:** Xiong Guo, Ling Liu, Qi Zhang, Weiming Yang, Yang Zhang

**Affiliations:** ^1^ Department of Colorectal and Anal Surgery, Hepatobiliary and Enteric Surgery Center, Xiangya Hospital, Central South University, Changsha, China; ^2^ Hepatobiliary & Enteric Surgery Research Center, Xiangya Hospital of Central South University, Changsha, China

**Keywords:** E2F7, USP47, MAPK, colon cancer 3, microRNA-199b

## Abstract

microRNAs (miRNAs) can modulate the expression level of genes in a post-transcription manner, which are closely related to growth and metastasis of colon cancer. Herein, we aimed to explore how miR-199b influences colon cancer and to characterize its underlying molecular mechanism associating with E2F transcription factor 7 (E2F7). Assays of RT-qPCR, Western blot, and immunohistochemistry were utilized to detect the expression of E2F7 in the tissue samples collected from 30 patients diagnosed with colon cancer. Flow analysis was utilized to detect the ratio of ALDH1^+^ and CD133^+^ colon cancer stem cells. The interaction between E2F7, miR-199b, USP47, and MAPK was identified by ChIP-Seq analysis, luciferase reporter, RNA pull-down, co-immunoprecipitation, as well as glutathione-S-transferase (GST) pull-down experiments. Based on the gain- and loss-of-function approaches, the cellular functions of colon cancer cells by the E2F7-regulated miR-199b/USP47/MAPK axis were assessed. It was identified that E2F7 are expressed highly in the collected colon cancer tissues. E2F7 silencing reduced the production of ALDH1^+^ and CD133^+^ colon cancer stem cells and antagonized the effects of 5-fluorouracil (5-FU) treatment. Besides, the silencing of E2F7 was observed to suppress the oxidative stress, proliferation, migration, as well as invasion of ALDH1^+^ cells *in vitro* and tumorigenesis of colon cancer cells *in vivo*. Our findings reveal the pro-oncogenic effect of E2F7 on colon cancer development, highlighting E2F7 as a novel target for therapeutic strategy for colon cancer.

## Introduction

Colon cancer has long been recognized as a common cancer type, and most patients with colon cancer suffer from severe cancer metastasis arising from migration of detached primary tumor cells to adjacent tissues or distant sites (1). Colon cancer often develops from regional adenomas in the intestinal epithelium, which convert to malignant tumors with an invasive nature that are likely to migrate to the liver (2). Targeting cancer stem cells has been regarded as a promising method for improving the prognosis of colon cancer patients (3). However, although great efforts have been made in improving clinical care and therapy modalities, the mortality remains extremely high in patients with colon cancer, especially those at advanced stages (4). Thus, there exists a need to explore the molecular mechanism underlying colon cancer to develop novel strategy options for colon cancer treatment.

E2F transcription factor 7 (E2F7) exerts tumor suppressive role by suppressing the transcription level of genes that participated in the entry of S-phase and disease progression (5). E2F7 is among the potential targets for molecular-based treatment of colon cancer patients (6). E2F7 modulates transcription and maturation of various microRNAs (miRNAs), which could regulate gene expression post-transcriptionally, to inhibit cell proliferation (7). Obviously, miRNAs have been widely documented to function importantly in the progression of colon cancer (8, 9). A previous study uncovered the possible participation of miR-199b in several sorts of biological processes, including the proliferative capacity and migratory ability of cells (10). Moreover, miR-199b inhibition has been implicated in distant metastasis in colorectal cancer (11). However, the lack of investigation on the specific involvement of miR-199b in colon cancer attracted our attention, which was designed as the purpose of our study.

In mice modeled with colon cancer, deletion of ubiquitin-specific protease 47 (USP47) can impair the transcriptional activity of the target gene special AT-rich sequence binding protein 1 (SATB1), thus inhibiting the proliferative and migratory capacities, as well as tumorigenicity of colon cancer cells (12). Besides, it has been well-documented that the USP47 deubiquitin-modified mitogen-activated protein kinase (MAPK) axis could stabilize MAPK that participates in the regulation of colon cancer metastasis (13, 14). Interestingly, it has been also illustrated the enhancement of MAPK signaling pathway in colon cancer tumor stem cell activity (15). However, the relationship among E2F7, miR-199b, and USP47 in colon cancer is scantly reported in the literature. Thus, we conducted investigation on the correlation among E2F7, miR-199b, and USP47 and their effects on colon cancer stem cell activity.

## Materials and Methods

### Ethics Statement

All experiments involving human beings and animals were carried out with the approval of the Ethics Committee and the Animal Management Committee of Xiangya Hospital of Central South University. Besides, the detailed experimental procedures were strict in line with the *Declaration of Helsinki* and the *Guide for the Care and Use of Laboratory Animals* published by the US National Institutes of Health. Prior to sample collection, every participant signed the documents of informed written consent.

### Microarray-Based Gene Expression Analysis

Through the GEPIA2 database, differentially expressed gene data of colon cancer was obtained, which included 275 colorectal cancer tissue and 41 normal tissue samples in the TCGA database and 308 normal tissue samples in the GTEx database. The motif of E2F7 of transcription factors was analyzed through the cistromeDB database. The relationship between transcription factor E2F7 and miR-199b was predicted by the hTFtarget database. The databases of StarBase, miDIP, and DianaTools were applied for prediction of the potential genes targeted by miRNA, and by combining with the low-expressed genes in colon cancer tissues in the GEPIA2 database, we obtained the intersection to identify the candidate target gene set of miR-199b.

### Study Subjects

In this study, 30 pairs of colon cancer and the corresponding adjacent normal tissues from the patients diagnosed with colon cancer (mean age: 44.63 ± 7.89 years) who underwent surgery at Xiangya Hospital of Central South University from June 2015 to June 2016 were collected. The patients were enrolled based on the inclusion criteria as follows: the nodules were determined as being colon cancer by pathological examination; no anti-cancer treatment was given before surgery; no tumor tissues on the surface were removed by pathological examination to confirm the complete resection of all tumor nodules; complete clinical pathology and follow-up data were provided. Patients dying from non-colonic disease or accident or those not agreeing to participate in a case–control study were excluded. The basic patient information was collected in a medical record facility, and all patients were followed up to determine in detail their post-treatment response and clinical outcomes. The follow-up lasted for 3–30 months, starting from the end of the surgery and ending in January 2020. Next, Kaplan–Meier was applied for analyzing the relationship of E2F7 expression with survival rate of patients.

### Culture of Colon Cancer Cells and ALDH^+/−^ Cells

Human colon cancer cell lines (SW620 and SW403) were obtained from the American Type Culture Collection (ATCC; Manassas, VA, USA), and ALDH^+/−^ cells were sorted by Aldefluor assay. Cells were cultured at 37°C with 5% CO_2_ with Dulbecco’s modified Eagle’s medium (DMEM) with the supplementation of 10% fetal bovine serum (FBS; 16,000,044) and 100 mg/L penicillin/streptomycin (15,070,063). All of these culture materials were purchased from Gibco-Invitrogen (Waltham, MA, USA).

### Lentiviral Transfection to Establish Stably Transfected Cell Lines

The short hairpin RNA (shRNA) sequence was inserted into the lentiviral shRNA fluorescent expression vector pSIH1-H1-copGFP to construct a gene knockout expression, and the MAPK sequence was constructed into the overexpression packaging vector phage-puro-6tag. Lentiviral vectors were completed by Gene Pharma (Shanghai, China) including shE2F7, shUSP47, and the negative control (NC) (sh-NC) (sequences: shE2F7 #1: GCTGCCAGCCCAGATATAAGG; shE2F7 #2: GCTATCCAAGTTATCCCTTGT; shUSP47 #1: GGTGCAGAAAGAGAGAGAGTT; shUSP47 #2: GCAGAAAGAGAGAGAGTTGGA). The 293T cells for lentivirus packaging were cultured in 10% FBS-contained DMEM, which were then passaged every other day. After that, the collected viruses were transfected with pSIH1-H1-copGFP-sh-NC, pSIH1-H1-copGFP-shE2F7, pSIH1-H1-copGFP-shUSP47, and pSIH1-H1-copGFP-shE2F7 + phage-puro-6tag-MAPK. The SW403 and SW620 cells cultured to the exponential phase were trypsinized, followed by preparation of cell suspension (5 × 10^4^ cells/ml) and overnight inoculation in a 6-well plate (2 ml/well) at 37°C. Next, the cells were subjected to a 48-h infection with the viruses (1 × 10^8^ TU/ml), followed by fluorescence microscopic observation on the expression efficiency of green fluorescent protein (GFP). The cells transfected with pSIH1-H1-copGFP-shE2F7 + phage-puro-6tag-MAPK were screened by further culture medium containing puromycin (1:10,000, 1,938,660, Gibco-Invitrogen, Waltham, MA, USA) to obtain stably transfected cell lines.

### Immunohistochemistry

Clinical tissue specimens were subjected to paraffin-embedding, xylene dewaxing, and alcohol gradient hydration and to subsequent 20-min immersion in 3% methanol–H_2_O_2_ for blocking the endogenous peroxidase. Following antigen retrieval, the sections were added with normal goat serum blocking solution (C-0005, Shanghai Haoran Biological Technology Co., Ltd, Shanghai, China) at room temperature for 20 min and with rabbit anti-human E2F7 (ab56022, 1:200, Abcam Inc., Cambridge, UK) overnight at 4°C. Following this, secondary antibody goat anti-rabbit immunoglobulin G (IgG) (ab6785, 1:1,000, Abcam Inc., Cambridge, UK) was added in the sections for culturing at 37°C for 20 min, followed by adding with horseradish peroxidase (HRP)-labeled streptavidin protein solution (0343–10,000 U, Imunbio Technology Co., Ltd, Beijing, China), and being allowed to stand at 37°C for 20 min. Thereafter, the sections were developed with diaminobenzidine (DAB) (ST033, Guangzhou Whiga Technology Co., Ltd., Guangzhou, China), counterstained with hematoxylin (PT001, Shanghai Bogoo Biotechnology Co., Ltd., Shanghai, China) for 1 min, blued in 1% ammonia, dehydrated with gradient alcohol, cleared in xylene, and sealed with neutral resin. Finally, the sections were observed and photographed under a microscope in five randomly selected high-power fields from each section.

### Reverse Transcription Quantitative Polymerase Chain Reaction

Total RNA was extracted using TRIzol reagents (15596026, Invitrogen, Carlsbad, CA, USA), and then reverse transcribed into complementary DNA by PrimeScript RT reagent Kit (RR047A, Takara, Japan). Then RT-qPCR was performed on an ABI PRISM 7300 real time quantitative PCR instrument (Applied Biosystems, Foster City, CA, USA) and Fast SYBR Green PCR Kit (Applied Biosystems, Foster City, CA, USA), with three repeats for each sample. Glyceraldehyde-3-phosphate dehydrogenase (GAPDH) was used as an internal reference to analyze the relative expression of target genes using the 2-ΔΔCt method. The primer sequences are shown in **Table 1**.

**Table 1:** Primer sequences for RT-qPCR.

Target	Primer sequences
miR-199b	Forward: 5′-CCCAGTGTTTAGACTATCTGTTC-3′
E2F7	Forward: 5′-GGAAAGGCAACAGCAAACTCT-3′
	Reverse: 5′-TGGGAGAGCACCAAGAGTAGAAGA-3′
USP47	Forward: 5′-CAACTGGTCCCGAAAGAGG-3′
GAPDH	Forward: 5′-CCCCGGTTTCTATAAATTGAGC-3′
	Reverse: 5′-CACCTTCCCCATGGTGTCT-3′
U6 RNA	Forward: 5′-CTCGCTTCGGCAGCACA-3′
	Reverse: 5v-AACGCTTCACGAATTTGCGT-3′

RT-qPCR, reverse transcription quantitative polymerase chain reaction; miR-199b, microRNA-199b; GAPDH, glyceraldehyde-3-phosphate dehydrogenase.

### Western Blot Analysis

The cells were collected, washed with phosphate-buffered saline (PBS), and lysed with protein lysate containing protease and alkaline phosphatase inhibitor, followed by incubation at 4°C for 30 min. Next, the cell lysate was collected into a 1.5 ml Eppendorf (EP) tube and centrifuged at 10,000 r/min for 15 min, with the supernatant harvested. A bicinchoninic acid (BCA) protein assay kit (Guangzhou GBCBIO Technologies Inc., Guangzhou, China) was then used to determine the protein concentration. After separation with 10% polyacrylamide gel electrophoresis, the protein was electro-transferred onto polyvinylidene fluoride (PVDF) membranes (0.3 A, 20 V). The membrane was blocked with 5% skimmed milk powder for 1 h, added with primary rabbit antibodies diluted with Tris-buffered saline Tween-20 (TBST) (E2F7, ab56022, 1:1,000; USP47, ab72143, 1:1,000; GAPDH, EPR16891, 1:3,000; K48 ubiquitin, EP8589, 1:1,000; FLAG, ab205606; HA, ab187915, Abcam Inc., Cambridge, UK; MAPK, and p44/42 MAPK [Erk1/2; #9102, Cell Signaling Technologies, Beverly, MA, USA]), and incubated overnight at 4°C. After washing three times with TBST, the membrane was re-probed with HRP-labeled secondary antibody, washed six times with TBST, and developed by enhanced chemiluminescence (ECL) reagents. The gel image analysis software Image J was used to analyze the gray value of each band. The ratio of the gray value of the target band to GAPDH was representative of the relative protein expression.

### Co-Immunoprecipitation Assay

The cells were collected, washed with PBS, added with 1 ml of protein lysate containing protease and alkaline phosphatase inhibitor, and incubated at 4°C for 30 min. Then the cell lysate was collected into a 1.5 ml EP tube and centrifuged at 10,000 r/min for 15 min to isolate the supernatant. The protein concentration was measured by the BCA method, and 1 mg of protein was taken from each sample and made up to 500 μl with IP lysis solution. The samples were added with corresponding antibody (FLAG, ab1162, Abcam Inc., Cambridge, UK; MAPK, p44/42 MAPK [Erk1/2], #9102, Cell Signaling Technologies, Beverly, MA, USA) and Protein A & G agarose and incubated at 4°C for 2–4 h. After that, the centrifugation pellet was washed three times with 1 ml lysis buffer, and then Western blot analysis was performed as above.

### Glutathione-S-Transferase Pull-Down Assay

BL21 competent cells were transformed with plasmids encoding glutathione-S-transferase (GST) and GST-USP47 and added with IPTG (1 mM) at 18°C for 16 h. Cells were lysed with lysis buffer (20 mM Tris-HCl, 200 mM NaCl, 5% glycerol, and 0.3% Triton X-100), then passed through an affinity chromatography column (Beijing Transgen Biotech Co., Ltd., Beijing, China) containing GST matrix, and finally eluted with glutathione peptide (10 mM in 50 mM Tris-HCl), which gave the purified protein. 293T cells were transfected with FLAG-MAPK, lysed with lysate, and then incubated with FLAG antibody (FLAG, ab1162, Abcam Inc., Cambridge, UK) and protein G agarose at 4°C for 2–4 h. After that, 3xFLAG peptide (100 mg/ml in PBS) (Sigma-Aldrich Chemical Company, St. Louis, MO, USA) was eluted to obtain FLAG-MAPK. GST/GST-USP47 (5 µg) was incubated with FLAG-MAPK at 4°C overnight, after which glutathione agarose was pulled down, and eluted three times before carrying out Western blot analysis.

### RNA Pull-Down Assay

Biotin-labeled RNAs were prepared *in vitro* using Biotin RNA Labeling Mix (Roche, Diagnostics, Basel, Switzerland) and T7 RNA polymerase (Promega, Madison, WI, USA) treated with RNase-free DNase I (Promega) and purified with Rneasy Mini Kit (Qiagen company, Hilden, Germany). Then, 3 g of biotin labeled RNA was heated to 90°C for 2 min, placed on ice for 2 min, added with RNA structure buffer (10 mM Tris, pH 7.0, 0.1 M KCl, 10 mM MgCl_2_), and allowed to stand at room temperature for 20 min. GST/GST-USP47 and biotin-labeled miR-199b were incubated in bicarbonate buffer at room temperature for 1 h. Streptavidin M280 beads (Thermo Fisher Scientific, Waltham, MA, USA) and RNA–protein mixture were incubated together at 4°C for 2–4 h, eluted three times and subjected to Western blot analysis.

### Chromatin Immunoprecipitation Assay

Chromatin immunoprecipitation assay (ChIP) kit (Merck Millipore, Billerica, MA, USA) was applied in the experiment. In brief, cells of each group were taken and upon reaching 70–80% confluence and were fixed in 1% formaldehyde at room temperature for 10 min to immobilize the DNA and protein in the cells. After cross-linking, the cells were randomly broken into fragments of an appropriate size by ultrasonic treatment and centrifuged at 13,000 rpm at 4°C with collection of the supernatant. The fragments were then divided into three tubes, added with positive control antibody RNA polymerase II, NC antibody normal human IgG (2729#, Cell Signaling Technologies, Beverly, MA, USA) and rabbit anti-E2F7 (1: 1000, ab56022, Abcam Inc., Cambridge, UK) respectively, and incubated overnight at 4°C. Protein Agarose/Sepharose was used to precipitate endogenous DNA–protein complexes, and after brief centrifugation, the supernatant was discarded. The non-specific complexes were washed, and cross-linking was decomposed overnight at 65°C. DNA fragments were purified by phenol/chloroform extraction, and RT-qPCR was used to check the expression of miR-199b promoter.

### Dual Luciferase Reporter Gene Assay

The promoter sequence, gene sequence of USP47, and the complete sequence of miR-199b were obtained through the NCBI database (http://www.ncbi.nlm.nih.gov/gene). The full length of the 3′untranslated region (3′UTR) of the amplified gene of USP47 was cloned. The PCR product was cloned into the multiple cloning site of downstream of the Luciferase gene of pGL3-control vector using the endonuclease sites XhoI and BamH I to construct pGL3-USP47-wild type (Wt) (USP47-Wt). The target gene database predicted the binding site of miR-199b and USP47, and the corresponding mutation site sequence was synthesized to construct pGL3-USP47-mutant type (Mut) vector (USP47-Mut). SW403 cells in the logarithmic growth phase were seeded in 6-well plates. After 24 h of routine culture, cells were transfected with miR-199b-NC and USP47-Wt, miR-199b-NC and USP47-Mut, miR-199b and USP47-Wt, as well as miR-199b and USP47-Mut. After 48 h, the cells were collected and lysed, centrifuged for 3 to 5 min, and the supernatant was analyzed using a luciferase detection kit (RG005, Shanghai Beyotime Institute of Biotechnology, Shanghai, China) according to the manufacturer’s instructions. Samples (20–100 μl for each group) were added with 100 μl firefly luciferase detection agent and mixed well with reporter cell lysate as a blank control, and placed in a Luminometer TD-20/20 fluorescence analyzer (E5311, Promega). The relative luciferase (RLU) activity was measured with renilla luciferase (Renilla) expression vector pRL-TK (TaKaRa) used as an internal reference. The RLU activity was calculated as the RLU activity of firefly luciferase/RLU activity of renilla luciferase.

### 5-Ethynyl-2′-Deoxyuridine Assay

Cells were seeded in a 24-well plate with three duplicate wells for each sample. 5-ethynyl-2′-deoxyuridine (EdU) was added to the culture solution to a concentration of 10 µM and incubated for 2 h. The medium was aspirated, fixed with PBS solution containing 4% paraformaldehyde for 15 min at room temperature, washed twice with PBS containing 3% bovine serum albumin (BSA), added with 0.5% Triton-100 in PBS and incubated at room temperature for 20 min. After washing twice with 3% PBS in BSA, each well was added with 100 µl of staining solution, incubated at room temperature in the dark for 30 min, and added with 4′,6-diamidino-2-phenylindole (DAPI) to stain the nucleus for 5 min. After mounting, the cells were observed in 6–10 randomly selected fields under a fluorescence microscope (FM-600, Shanghai Puda Optical Instrument Co., Ltd., Shanghai, China), and the number of positive cells was recorded in each field. EdU labeling rate (%) = number of positive cells/(number of positive cells + number of negative cells) × 100%.

### Scratch Test

At the bottom of the 6-well plate, a ruler and a marker were used to draw at least five horizontal lines separated by 0.5–1 cm. The 6-well plate was added with about 5 × 10^5^ cells/well and incubated overnight in medium containing 10% fetal bovine serum. Then, sterile 10 µl pipette tips were used to make scratches perpendicular to the back horizontal line, and the distance of the scar was measured and recorded under an optical microscope after incubation for 0 and 24 h. Then, images were recorded under an inverted microscope to observe the cell migration status of each group.

### Transwell Assay

The apical chamber surface of the bottom membrane of the Transwell chamber was coated with Matrigel of BD Company, and the Matrigel was polymerized into a gel at 37°C for 30 min, and the basement membrane was hydrated before use. The cells were cultured in serum-free medium for 12 h, harvested and resuspended in serum-free medium (1 × 10^5^/ml). The culture containing 10% fetal bovine serum was added in the basolateral chamber. Then, 100 µl of the cell suspension was added to the Transwell chamber. After incubation at 37°C for 24 h, the cells failing to invade the surface of the Matrigel membrane were gently removed with a cotton swab, fixed with 100% methanol, and stained with 1% toluidine blue (Sigma-Aldrich Chemical Company, St Louis, MO, USA). The stained invasive cells in five randomly selected fields were counted under an inverted light microscope (Carl Zeiss, Germany).

### Tumor Formation in Nude Mice

Six-week-old specific pathogen-free (SPF) nude mice were classified into two groups (n = 10). Then the right posterior ribs were subcutaneously injected with transfected cells (5 × 10^6^ cells per 100 µl, diluted in PBS) to monitor tumor growth for about six weeks, and the mice were then euthanized for follow-up analysis.

### Aldefluor Assay and ALDH^+/−^ Cell Sorting

The Aldefluor kit (Stem Cell Technologies, Vancouver, BC, Canada) was used to determine the proportion of high ALDH enzyme viable cells. In the procedure, 1 × 10^6^ cells in each group were resuspended n Aldefluor assay buffer containing ALDH substrate. After incubation at 37°C, the cells were centrifuged and resuspended in 500 µl Aldefluor buffer and then analyzed with Navios flow cytometer, where green fluorescent FL1 analysis indicated the number of ALDH^+^ cells.

### Sphere Formation Assay

Colon cancer cells were seeded into an ultra-low-attachment plate (Corning, Bedford, MA, USA) at a density of 2,000 cells/ml and cultured in serum-free DMEM containing 20 ng/ml epidermal growth factor (Sigma-Aldrich Chemical Company, St Louis, MO, USA), 20 ng/ml basic fibroblast growth factor (Sigma-Aldrich Chemical Company, St Louis, MO, USA), and 5 μg/ml insulin (Sigma-Aldrich Chemical Company, St Louis, MO, USA). After 5–7 days of culture, these cells could form stem-like cell aggregates, and the number of tumor spheres formed was counted under a XSP-44 microscope (Olympus Optical Co., Ltd, Tokyo, Japan).

### Flow Cytometry

Colon cancer cells were detached with accutase enzyme (Gibco, Grand Island, NY, USA), neutralized with culture medium, centrifuged at 300 g for 5 min, and then re-suspended in 500 μl staining buffer. The cell suspension was put into new flow tubes, centrifuged at 300 g for 5 min, and then re-suspended in 500 μl staining buffer. Next, the suspension was added with phycoerythrin-coupled rabbit antibody to CD133 (ab19898, 1:1,200, Abcam Inc., Cambridge, UK), mixed, and incubated at 4°C for 30 min. Following 5 min of centrifugation at 300 g, the pellet was washed twice with 500 μl staining buffer, re-suspended with 200–300 μl staining buffer and analyzed with a BD FACSCalibur flow cytometer (BD Bioscience; San Jose, CA, USA).

### Statistical Analysis

Data statistical analyses were processed using the Statistic Package for Social Science (SPSS) 21.0 statistical software (IBM Corp. Armonk, NY, USA). Measurement data were expressed as mean ± standard deviation. Conforming to normal distribution and homogeneity of variance, paired data between intestinal cancer tissues and adjacent normal tissues were compared using *t-*test, while unpaired data between the other two groups were analyzed using unpaired *t*-test. Comparisons among multiple groups were conducted by one-way analysis of variance (ANOVA) followed by Tukey’s *post hoc* test. The Kaplan–Meier method was used to calculate the survival rate of patients, and log-rank test was used for univariate analysis. A value of *p* < 0.05 was considered indicative of statistical significance.

## Results

### E2F7 Was Highly Expressed in Colon Cancer Tissues and Was Inversely Related to the Survival Rate of Patients

**Figure 1:**
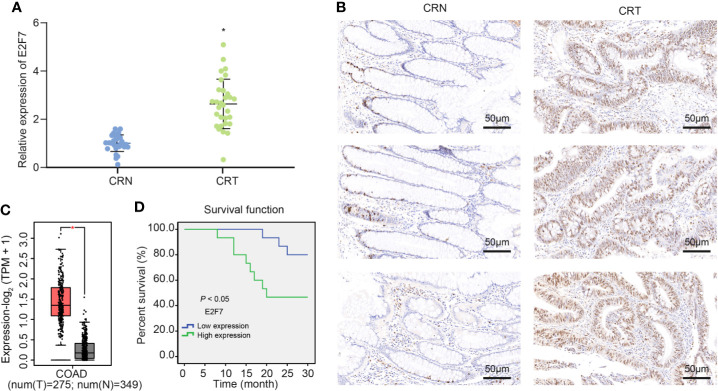
E2F7 is overexpressed in colon cancer tissues and negatively correlates with the survival rate of patients. **(A)** E2F7 expression in cancer tissues and adjacent normal tissues collected from 30 patients with colon cancer determined by RT-qPCR. The Y axis indicates the relative expression of E2F7 mRNA. **(B)** Immunohistochemical staining analysis of E2F7 protein in three pairs of colon cancer tissues and adjacent normal tissues (×200). **(C)** The expression of E2F7 in colon cancer tissues of the GEPIA2 database, where red indicates tumor samples and gray indicates normal tissues. The Y axis indicates the relative expression of E2F7 mRNA. **(D)** Correlation of survival rate of patients with E2F7 expression analyzed by a survival curve. X axis indicates time, and Y axis indicates survival rate. *p < 0.05. Data between colon cancer tissues and adjacent normal tissues were compared using paired *t*-test. The experiment was repeated three times independently.

A previous literature reported the possible involvement of E2F7 in the colon cancer initiation and progression (16). E2F7 expression examined by RT-qPCR showed an enhancement in the tumor tissue samples from 30 patients with colon cancer (**Figure 1A**). Meanwhile, immunohistochemistry revealed an increased expression of E2F7 protein in the colon cancer tissues (**Figure 1B**). Analysis using the GEPIA2 database also suggested upregulation of E2F7 expression in colon cancer tissues (**Figure 1C**). As Kaplan–Meier survival analysis revealed, the colon cancer patients with high expression of E2F7 had a lower survival rate in (**Figure 1D**). Therefore, it suggested an upregulated E2F7 in colon cancer tissues, which was associated with low survival rate of patients.

### Silencing E2F7 Repressed the Stemness of Colon Cancer Stem Cells

**Figure 2:**
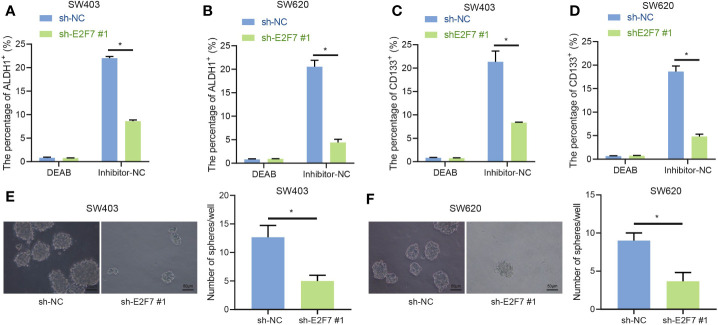
E2F7 silencing suppresses the stemness of colon cancer stem cells. **(A)** Flow cytometry and quantitative analysis of the proportion of ALDH1^+^ cells in SW403 cells transfected with shE2F7. **(B)** Flow cytometry and quantitative analysis of the changes in the proportion of ALDH1^+^ cells in SW620 cells transfected with shE2F7. **(C)** Flow cytometry and quantitative analysis of the proportion of CD133+ cells in SW403 cells transfected with shE2F7. **(D)** Flow cytometry and quantitative analysis of the proportion of CD133+ cells in SW620 cells transfected with shE2F7. **(E)** SW403 cell stemness determined by sphere formation test upon transfection with shE2F7. **(F)** SW620 cell stemness determined by sphere formation test upon transfection with shE2F7. Data between two groups were compared using unpaired *t*-test. *p < 0.05. The experiment was repeated three times independently.

According to previous literature reports, the important involvement of ALDH1^+^ colon cancer stem cells has been implicated in colon cancer (15). Thus, we speculated that promotion of E2F7 expression would occur in ALDH1^+^ colon cancer stem cells. Diethylaminobenzaldehyde (DEAB) (ALDH inhibitor), which was used as the control condition to distinguish the ALDH1^+^ cell population in the human colon cancer cell lines SW403 and SW620 and accurately analyze the altered number and proportion of ALDH1^+^ cells induced by E2F7, obviously inhibited the proportion of ALDH1^+^ cells. In SW403 and SW620 cells, the proportion of ALDH1^+^ cells was much higher in response to sh-NC + inhibitor-NC treatment relative to shE2F7 + inhibitor-NC treatment, while in cells transfected with sh-NC + DEAB or shE2F7 + DEAB, the lowest proportion of ALDH1^+^ cells was observed (**Figures 2A, B** **)**. Besides, the influence E2F7 has on CD133+ colon cancer stem cells was examined, finding results consistent with those from ALDH1^+^ cells (**Figures 2C, D** **)**. All in all, E2F7 silencing decreased the proportion of ALDH1^+^ and CD133+ colon cancer tumor stem cells. Furthermore, the sphere formation assay revealed that E2F7 knockdown attenuated the stemness of colon cancer tumor stem cells (**Figures 2E, F** **)**. These results supported the promoting effect of E2F7 on the stemness of colon cancer tumor stem cells.

### E2F7 Silencing Inhibited Antagonistic Effect of ALDH1^+^ Cells on 5-Fluorouracil Treatment and Oxidative Stress

**Figure 3:**
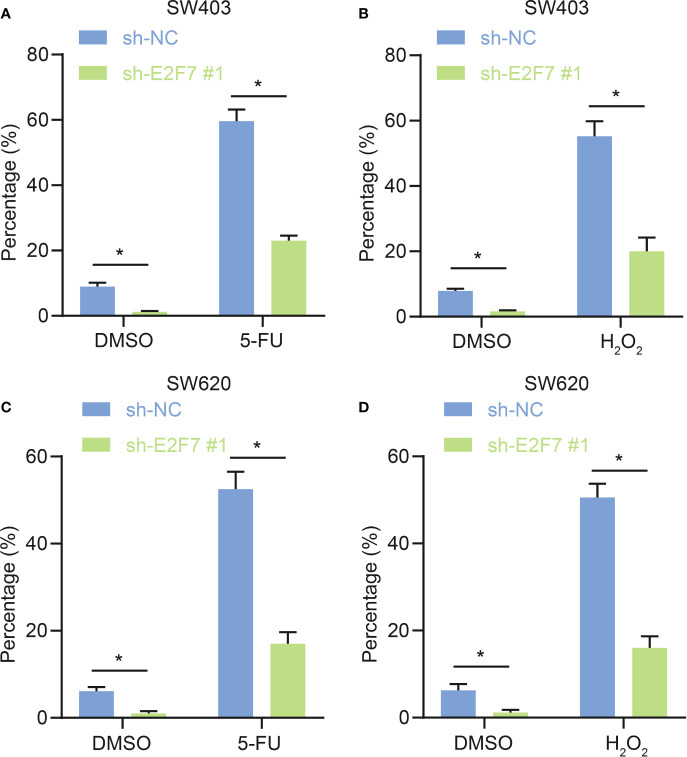
E2F7 silencing represses antagonistic effects of ALDH1^+^ cells on 5-FU treatment and oxidative stress. **(A)** In SW403, flow cytometry and quantitative analysis of the effect of E2F7 silencing on antagonism of ALDH1^+^ cell to effects of 5-FU treatment. **(B)** In SW403 cells, flow cytometry and quantitative analysis of the effect of E2F7 silencing on antagonism of ALDH1^+^ cells to H_2_O_2_ treatment. **(C)** In SW620 cells, flow cytometry and quantitative analysis of the effect of E2F7 on ALDH1^+^ cell antagonist 5-FU. **(D)** In SW620 cells, flow cytometry and quantitative analysis of the effect of E2F7 on ALDH1^+^ cells antagonizing H_2_O_2_ treatment. Data between two groups were compared using unpaired *t*-test. *p < 0.05. The experiment was repeated three times independently.

The chemotherapeutic drug5-5-fluorouracil (FU) has been utilized clinically for colon cancer. The oxidative stress signaling pathway was activated in ALDH^+^ cells, and ALDH1^+^ cells were protected from 5-FU treatment and oxidative stress (15), leading us to predict that E2F7 might facilitate this process. In SW402 and SW620 cells, the proportions of ALDH1^+^ cells were lower upon treatment with shE2F7 + 5-FU relative to sh-NC + 5-FU (**Figures 3A, C** **)**. Moreover, there was a lower proportion of ALDH1^+^ cells after treatment with shE2F7 + H_2_O_2_ relative to sh-NC + H_2_O_2_ (**Figures 3B, D** **)**. It demonstrated that E2F7 silencing reduced the antagonistic effects of ALDH1^+^ cells on 5-FU treatment and oxidative stress.

### E2F7 Inhibited the Expression of miR-199b by Binding to its Promoter

**Figure 4:**
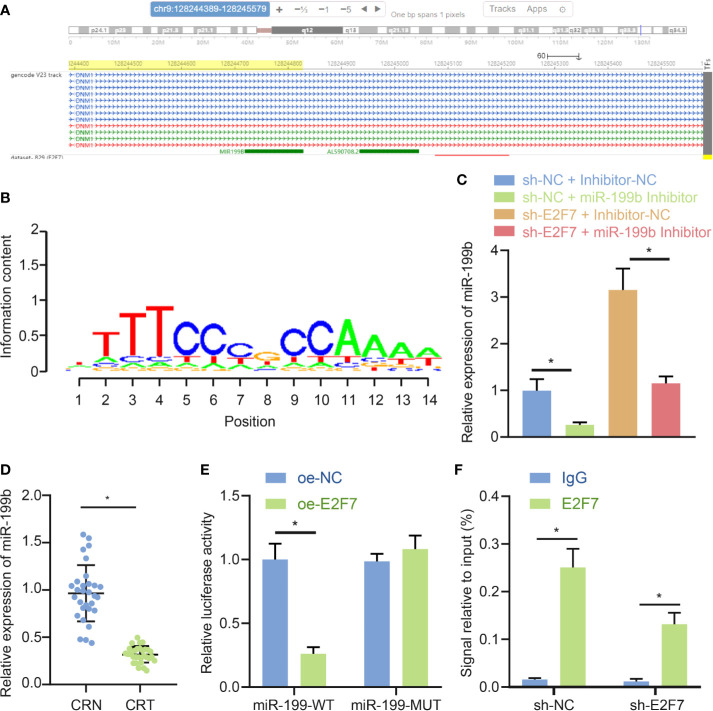
E2F7 reduces miR-199b expression by binding to the miR-199b promoter. **(A)** Analysis of Motif of E2F7 through the cistromeDB database. **(B)** Visualization of ChIP-seq results of miR-199b targeting E2F7 in the hTFtarget database. **(C)** Expression of miR-199b determined by RT-qPCR in SW403 cells transfected with shE2F7 or miR-199b inhibitor. **(D)** miR-199b expression in colon cancer tissues and adjacent normal tissues determined by RT-qPCR (n = 30). **(E)** Binding of E2F7 to the miR-199 promoter region confirmed by luciferase reporter gene assay. **(F)** Binding of E2F7 to the miR-199 promoter region analyzed by ChIP assay. Data between colon cancer tissues and adjacent normal tissues were compared using paired *t-*test, while data between other two groups were compared using unpaired *t*-test. Comparisons among multiple groups were conducted by one-way ANOVA followed by Tukey’s *post hoc* test. *p < 0.05. The experiment was repeated three times independently.

Previous literature reported an inhibitory role of E2F7 in the expression of its downstream genes (17). Motif of transcription factor E2F7 was obtained through analysis using the cistromeDB database (http://cistrome.org/db/#/) (**Figure 4A**). In addition, miR-199b-5p was determined to have low expression in colon cancer (11). Then whether the transcription factor E2F7 had a targeting relationship with miR-199b was predicated, and the results showed that E2F7 was enriched in the miR-199b promoter region (**Figure 4B**). In SW403 cells, silencing E2F7 promoted the expression of miR-199b, and this promotion was offset by miR-199b inhibitor (**Figure 4C**). Moreover, as suggested by RT-qPCR, miR-199b had low expression in colon cancer tissues (**Figure 4D**). Dual luciferase reporter assay uncovered that the activity of miR-199 promoter was decreased in response to overexpression of E2F7 (**Figure 4E**). Results of ChIP detection found that after silencing E2F7, E2F7 binding to the promoter of miR-199b was reduced, thereby promoting the expression of miR-199b (**Figure 4F**). Taken together, E2F7 decreased the expression of miR-199b by binding to the miR-199b promoter.

### miR-199b Targeted and Inhibited USP47 Expression in SW403 Cells

**Figure 5:**
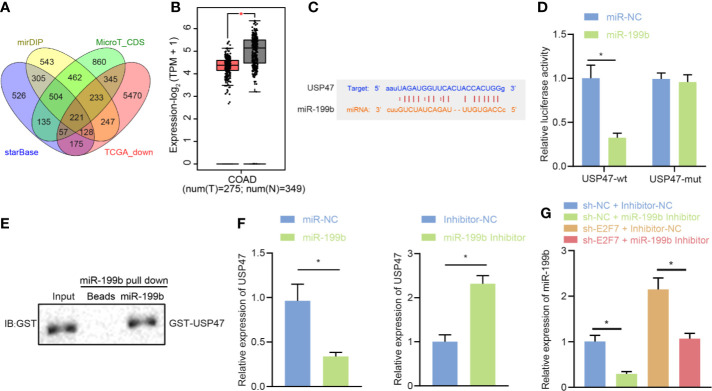
USP47 is a target gene of miR-199b in SW403 cells. **(A)** Prediction of lowly expressed target gene set of miR-199b by starBase, miDIP, and DianaTools. **(B)** The expression of USP47 in colon cancer tissues in the GEPIA2 database, where red indicates tumor samples and gray indicates normal tissues. **(C)** Putative miR-199b binding sites in the 3′UTR of UPS47 mRNA. **(D)** Binding of miR-199b to USP47 confirmed by dual luciferase reporter gene assay. **(E)** RNA pull-down analysis of the interaction between miR-199b and GST-USP47 *in vitro*. **(F)** USP47 expression determined by RT-qPCR and Western blot analysis in cells transfected with miR-199b or miR-199b inhibitor. **(G)** USP47 expression determined by RT-qPCR and Western blot analysis in cells transfected with shE2F7. Comparisons among multiple groups were conducted by one-way ANOVA followed by Tukey’s *post hoc* test. Data between two groups were compared using unpaired *t-*test. *p < 0.05. The experiment was repeated three times independently.

With the attempt to investigate the downstream mechanism of miR-199b, starBase, miDIP, and DianaTools databases were utilized for the prediction on the target genes of miR-199b. The obtained data was intersected with the poorly expressed genes in colon cancer tissues from the GEPIA2 database to identify the candidate target genes of miR-199b (**Figure 5A**). The expression of USP47 in colon cancer tissues from the GEPIA2 database showed a downward trend (**Figure 5B**). Further prediction through the starBase website showed the presence of binding sites between miR-199b and USP47 (**Figure 5C**). Dual luciferase reporter gene assay further implicated a weakened USP47-Wt luciferase activity and did not affect luciferase activity of USP47-Mut (*p* > 0.05) in SW403 cells after miR-199b mimic treatment (**Figure 5D**). Results of RNA pull-down assay exhibited an interaction of miR-199b with USP47 *in vitro* (**Figure 5E**). In SW403 cells, the assays of RT-qPCR and Western blot demonstrated a descended USP47 expression by overexpression of miR-199b and an ascended trend by miR-199b inhibitor (**Figure 5F**). Besides, it also revealed a reduced miR-199b expression and an elevated USP47 expression in the SW403 cells in response to the treatment of sh-NC + miR-199b inhibitor than the treatment of sh-NC + inhibitor-NC, while opposite results were revealed after the treatment with shE2F7 + inhibitor-NC. In addition, simultaneous knockdown of E2F7 and miR-199b led to a decline of miR-199b expression and an increase of USP47 expression in SW403 cells (**Figure 5G**). All the above-mentioned data declared the targeting regulatory relations between miR-199b and USP47. E2F7 upregulated USP47 expression by downregulating miR-199b in SW403 cells.

### E2F7 Upregulated USP47 Expression to Deubiquitinate the K48 Ubiquitin Chain of MAPK to Stabilize MAPK

**Figure 6:**
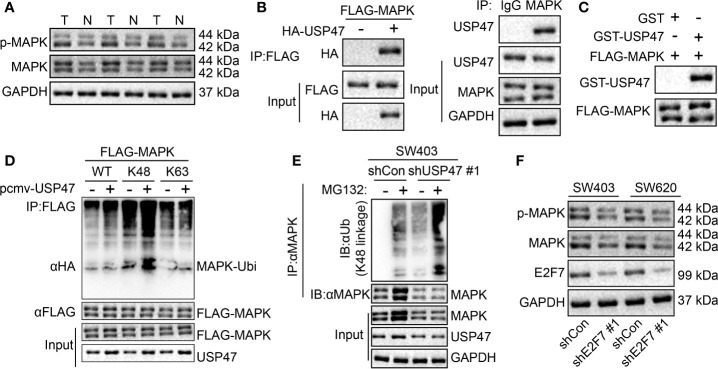
USP47 upregulation by E2F7 deubiquitin-modifies MAPK K48 ubiquitin chain to stabilize MAPK. **(A)** Protein expression of MAPK in three pairs of colon cancer tissues (T) and adjacent normal tissues (N) determined by Western blot analysis. **(B)** Detection of the interaction between USP47 and MAPK in 293T cells and SW403 cells. **(C)** GST pull-down analysis of the interaction between USP47 and MAPK *in vitro*. **(D)** In 293T cells, detection of the type of MAPK deubiquitin by USP47. **(E)** In SW403 cells, detection of MAPK deubiquitination. **(F)** MAPK expression determined by Western blot analysis in SW403 and SW620 cells transfected with shE2F7. The experiment was repeated three times independently.

A previous report suggested that USP47 deubiquitin-modified MAPK was stabilized (13). We found an increased MAPK expression and extent of MAPK phosphorylation in colon cancer tissues (**Figure 6A**). At the cellular level, interactions between USP47 and MAPK were detected in both overexpression and endogenous experiments (**Figure 6B**). The results of GST pull-down experiment displayed that USP47 directly interacted with MAPK *in vitro* (**Figure 6C**). Additionally, in 293T cells, USP47 was detected to be able to deubiquitinate the MAPK K48 instead of the ubiquitin type of K63 (**Figure 6D**). In SW403 cells, MAPK would accumulate, and K48 ubiquitination of MAPK increased following the MG132 treatment. Whereas after USP47 knock down, the background level of MAPK was decreased, and K48 ubiquitination was increased. Furthermore, MAPK did not accumulate after MG132 treatment, nor did K48 ubiquitination increase (**Figure 6E**). To further explore the regulation of E2F7 on MAPK, we knocked down E2F7 in SW403 and SW620 cells. Results of Western blot assay demonstrated a decrease in the MAPK protein level and MAPK phosphorylation extent in the absence of E2F7 (**Figure 6F**). These results showed that E2F7 regulated USP47 to deubiquitinate the MAPK K48 ubiquitin chain, thus stabilizing MAPK.

Moreover, the E2F7 expression was evaluated in common colon cancer cell lines, among which the LOVO colon cancer cells with the lowest E2F7 expression detected was selected for overexpression experiments. We found that the expression of USP47 and MAPK was increased following overexpression of E2F7, which was basically consistent with our conclusion (**Supplementary Figure 1**).

### E2F7 Upregulated MAPK to Promote Proliferation, Invasion, and Migration of Colon Cancer Cells In Vitro as Well as Accelerating Tumor Growth In Vivo

**Figure 7:**
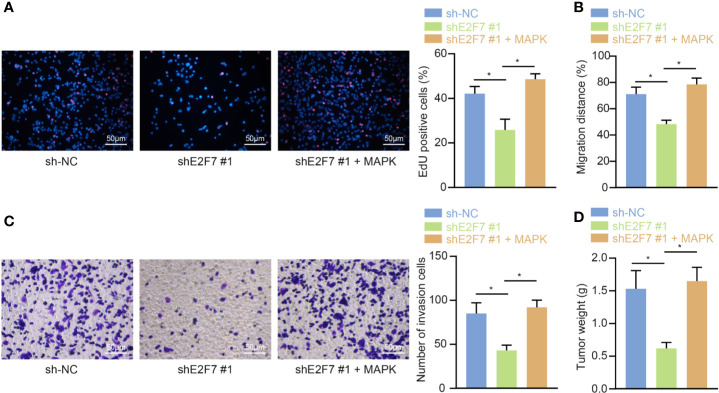
E2F7 promotes *in vitro* proliferation, invasion, and migration, and *in vivo* tumorigenesis of colon cancer cells by upregulating MAPK expression. **(A)** Detection of SW403 cell proliferation by EdU assay (×200). **(B)** Detection of the migration of SW403 cells by scratch test. **(C)** Detection of SW403 cell invasion by Transwell assay. **(D)** Tumor size and weight of mice (n = 10). Comparisons among multiple groups were conducted by one-way ANOVA followed by Tukey’s *post hoc* test. *p < 0.05. The experiment was repeated three times independently.

For the purpose of exploring whether E2F7 affects colon cancerous cell growth abilities, SW403 cells were subjected to the treatments of sh-NC, shE2F7, or shE2F7 + phage-MAPK. The results of the EdU experiment (**Figure 7A**), scratch test (**Figure 7B**), and Transwell assay (**Figure 7C**) exhibited that knockdown of E2F7 diminished the proliferative, invasive, and migratory abilities of the SW403 cells, and these effects were neutralized by dual treatment with shE2F7 and MAPK. The animals were then administrated with the injection of SW403 cells following transfection with sh-NC, shE2F7, or shE2F7 + phage-MAPK. Six weeks later, E2F7 knockdown reduced tumor volume and weight, which was negated following dual treatment with shE2F7 and MAPK (**Figure 7D**). The above-mentioned data uncovered the promotive effect of E2F7-activated MAPK axis on the proliferative, invasive, and migratory abilities of colon cancerous cells, along with the ability of tumorigenesis.

## Discussion

Colon cancer is regarded as one of the primary reasons of mortality associated with cancers the world over (18). The E2F family is a gene group encoding a series of transcription factors in higher eukaryotes, which may participate in mammalian cell cycle regulation and DNA synthesis (6). Findings obtained from a previous study suggest that E2F1 and E2F7 have different regulatory effects on KPNA2 to promote the development of gallbladder cancer (19). In the current research, the E2F family member E2F7 promoted the development of colon cancer. Collectively, the data of our experiments provided evidence that E2F7 transcriptionally inhibits miR-199b expression, and miR-199b targets USP47 that stabilizes MAPK through blocking ubiquitin-mediated degradation of MAPK, which subsequently enhances the malignant characteristics of colon cancerous cells.

Our initial results showed a high level of E2F7 in colon cancer tissues, while the inhibitory effect of E2F7 silencing was found on the production of ALDH1^+^ and the antagonistic effects of ALDH1^+^ cells on 5-FU treatment and oxidative stress in colon cancer tumor stem cells. A prior study revealed that E2F7 promoted the development of colon cancer (6). The expression of ALDH1 indicates stemness and is a biomarker in colon cancer (20). 5-FU is used clinically as a chemotherapeutic drug for colon cancer treatment (21). The involvement of ALDH1^+^ colon cancer stem cells was previously reported in colon cancer occurrence, and ALDH1^+^ cells could antagonize the effects of 5-FU treatment and oxidative stress (15).

In the subsequent experiments, we found promotion of E2F7 on the progression of colon cancer through its inhibition on miR-199b expression. It has been investigated that E2F7 could bind to its downstream gene promoter, thereby inhibiting the expression of downstream genes (17), and modulating transcription and maturation of numerous miRNAs in various cells (7). Ectopic E2F7 expression has been signified to inhibit the therapeutic effect of miR-26a in breast cancer cells (22). These findings indirectly support our present results that E2F7 inhibited miR-199b expression. Also in line with our data, a previous qualitative research revealed the regulation of miR-199b on the sirtuin 1/cAMP responsive element binding protein/kisspeptin (SIRT1/CREB/KISS1) signaling pathway and may thus influencing colon cancer (11). miRs are important gene regulatory factors that bind to target genes and inhibit their expression at the post-transcriptional expression (23). For example, miR-199b targets JAG1 to inhibit the proliferation of porcine muscle satellite cells (24). Taken together, the above evidence indirectly proved the result of the present investigation that USP47 is targeted by miR-199b. Ubiquitination is a cellular process that induces proteasome degradation by covalently linking ubiquitin to the substrate protein (25). Other lines of research indicate that abnormal histone ubiquitination can drive tumorigenesis by changing the expression of tumor suppressor genes and tumor suppressor genes, mis-regulating cell differentiation, and promoting cancer cell proliferation (26). Deubiquitinase USP47 has been reported to stabilize MAPK *via* its counteraction on the effect of n-terminal ligase POE/UBR4 on Drosophila (13). All in all, the results of this study revealed that E2F7 inhibited miR-199b to enhance the development of colon cancer by targeting USP47.

Additionally, our findings also revealed that E2F7 promoted MAPK stability to enhance cell proliferation, invasion, and migration in colon cancer. Similarly, a prior study elucidated the association of the MAPK signaling pathway with colon cancer growth *in vivo* (27). Notably, MAPK has been found to promote colon cancer tumor stem cell activity (15). In addition, it has been illustrated that MAPK and the AKT/MAPK signaling pathway could enhance the proliferative, migratory, as well as invasive capabilities of colon cancer cells (28, 29). A recent study also revealed the same promotive role of E2F7 in EC cellular processes (30). Therefore, we are convinced that present results show that E2F7 suppressed the miR-199b expression and promoted USP47 to stabilize MAPK proteins, thereby contributing to colon cancer development.

**Figure 8:**
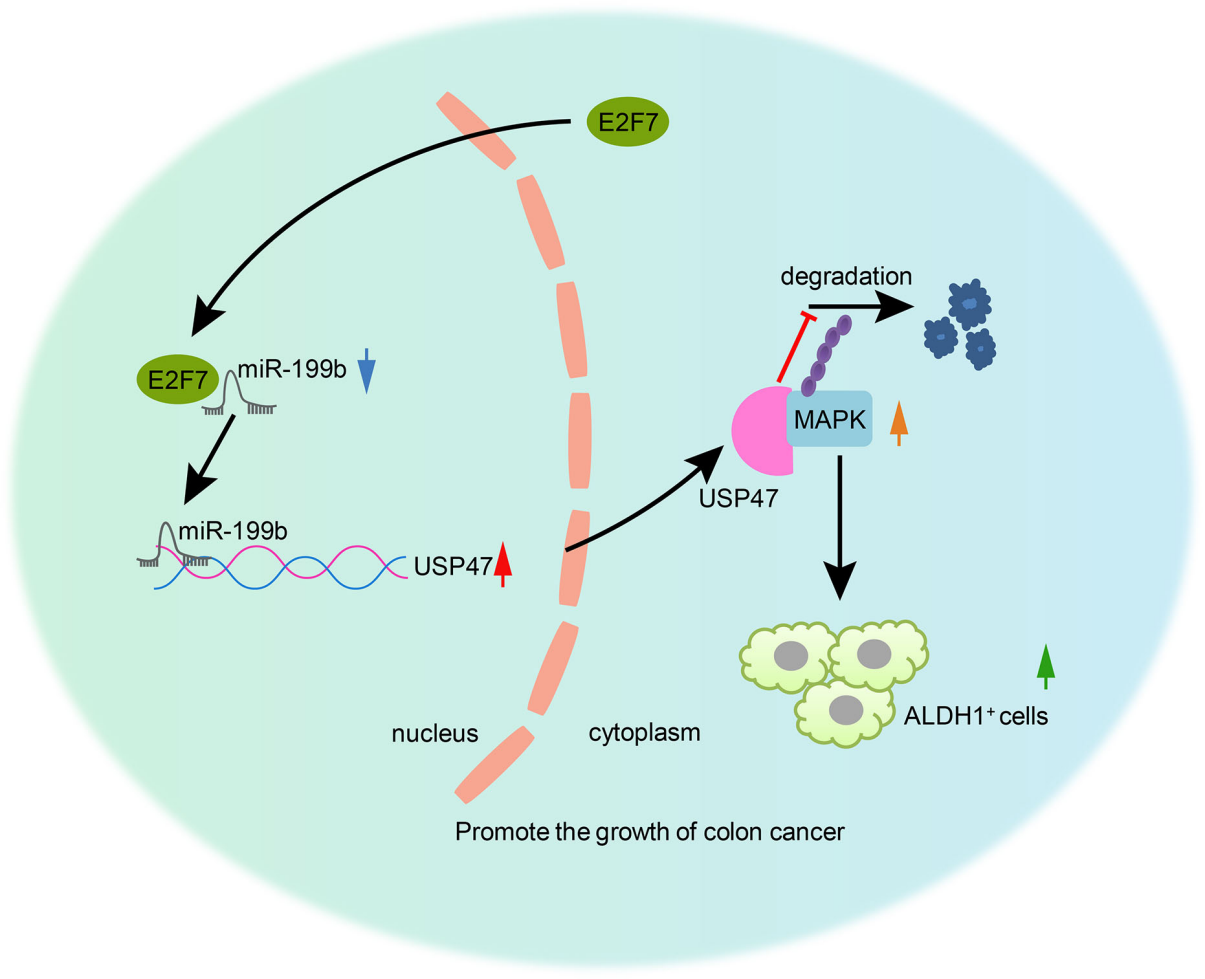
The mechanism of E2F7 in colon cancer. E2F7 inhibited miR-199b expression and consequently upregulated the miR-199b target USP47, thus stabilizing MAPK protein and inducing the increase of the cancer stem cell activity, which would then be responsible for the aggravation of colon cancer.

In conclusion, our study reported that E2F7 downregulated the expression of miR-199b, leading to the upregulation of the miR-199b target USP47 that stabilized MAPK, promoting colon cancer stem cell activity, thereby accelerating the development and progression of colon cancer (**Figure 8**). Therefore, miR-199b might be considered as a promising therapeutic approach for treating colon cancer. However, it remains to be established if present findings generalize to broader patient populations and cancer cell lines. Indeed, our study may eventually justify proposing clinical trials, pending a further elaboration of the effects of miR-199b in the pathway to colon cancer.

## Data Availability Statement

The raw data supporting the conclusions of this article will be made available by the authors, without undue reservation.

## Ethics Statement

The studies involving human participants were reviewed and approved by the Ethics Committee of Xiangya Hospital of Central South University. The patients/participants provided their written informed consent to participate in this study. The animal study was reviewed and approved by the Animal Management Committee of Xiangya Hospital of Central South University.

## Author Contributions

XG conceived and designed the research. LL performed the experiments and interpreted the results of the experiments. QZ analyzed the data and prepared the figures. WY drafted the paper. YZ edited and revised the manuscript. All authors contributed to the article and approved the submitted version.

## Conflict of Interest

The authors declare that the research was conducted in the absence of any commercial or financial relationships that could be construed as a potential conflict of interest.

## Supplementary Material

Supplementary Figure 1 Overexpression of E2F7 promotes the expression of USP47 and MAPK. Protein expression of E2F7, USP37, and MAPK1 as well as the extent of MAPK1 phosphorylation determined by Western blot analysis. Comparisons between two groups were conducted by unpaired *t*-test. The experiment was repeated three times independently.

## Abbreviations

miRNAs or miRs, microRNAs; E2F7, E2F transcription factor 7; USP47, ubiquitin-specific protease 47; SATB1, special AT-rich sequence binding protein 1; MAPK, mitogen-activated protein kinase; ATCC, American Type Culture Collection; DMEM, Dulbecco’s modified Eagle’s medium; FBS, fetal bovine serum; shRNA, short hairpin RNA; NC, negative control; GFP, green fluorescent protein; DAB, diaminobenzidine; RT-qPCR, reverse transcription quantitative polymerase chain reaction; IgG, immunoglobulin G; HRP, horseradish peroxidase; GAPDH, Glyceraldehyde-3-phosphate dehydrogenase; PBS, phosphate-buffered saline; EP, Eppendorf; BCA, bicinchoninic acid; PVDF, polyvinylidene fluoride; TBST, Tris-buffered saline Tween-20; HRP, horseradish peroxidase; ECL, enhanced chemiluminescence; GST, Glutathione-S-transferase; ChIP, Chromatin immunoprecipitation assay; 3′UTR, 3′untranslated region; Wt, wild type; Mut, mutant type; RLU, relative luciferase; EdU, 5-ethynyl-2′-deoxyuridine; DAPI, 4′,6-diamidino-2-phenylindole; FM, fluorescence microscope; SPF, specific pathogen-free; DEAB, Diethylaminobenzaldehyde; SPSS, Statistic Package for Social Science; ANOVA, analysis of variance; 5-FU, 5-fluorouracil; SIRT1/CREB/KISS1, sirtuin 1/cAMP responsive element binding protein/kisspeptin.
