# Supplementary figures and images for: E2F7 Transcriptionally Inhibits MicroRNA-199b Expression to Promote USP47, Thereby Enhancing Colon Cancer Tumor Stem Cell Activity and Promoting the Occurrence of Colon Cancer
Source: Front Oncol. 2021 Jan 7;10:565449. doi: 10.3389/fonc.2020.565449 (PMC7819137; doi:10.3389/fonc.2020.565449)

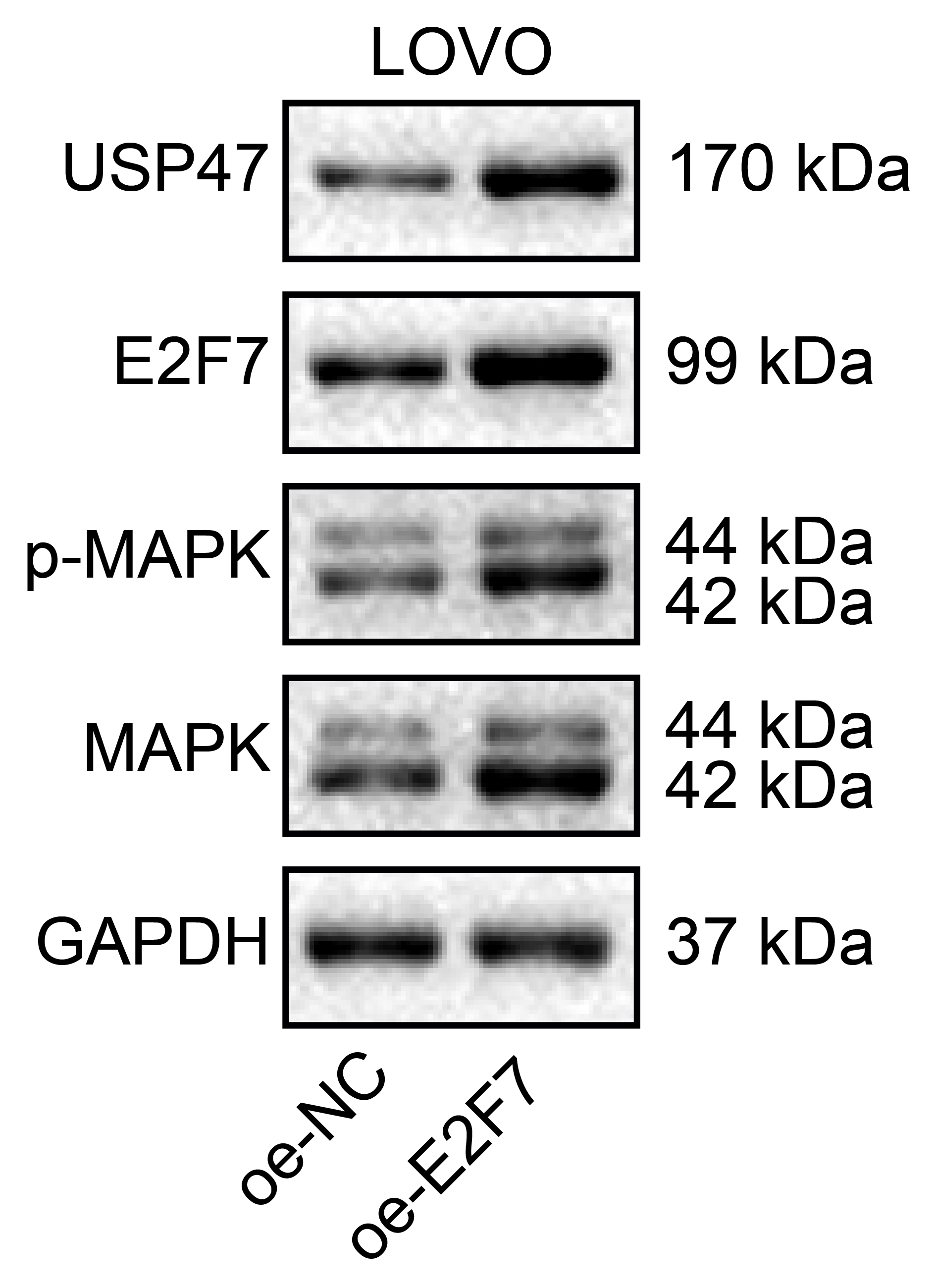

Supplement: Supplementary Figure 1 — Overexpression of E2F7 promotes the expression of USP47 and MAPK. Protein expression of E2F7, USP37, and MAPK1 as well as the extent of MAPK1 phosphorylation determined by Western blot analysis. Comparisons between two groups were conducted by unpaired t-test. The experiment was repeated three times independently. [file Image_1.jpeg]
